# PheNormGPT: a framework for extraction and normalization of key medical findings

**DOI:** 10.1093/database/baae103

**Published:** 2024-10-23

**Authors:** Ekin Soysal, Kirk Roberts

**Affiliations:** McWilliams School of Biomedical Informatics, University of Texas Health Science Center at Houston, 7000 Fannin St #600, Houston, TX 77030, United States; McWilliams School of Biomedical Informatics, University of Texas Health Science Center at Houston, 7000 Fannin St #600, Houston, TX 77030, United States

## Abstract

This manuscript presents PheNormGPT, a framework for extraction and normalization of key findings in clinical text. PheNormGPT relies on an innovative approach, leveraging large language models to extract key findings and phenotypic data in unstructured clinical text and map them to Human Phenotype Ontology concepts. It utilizes OpenAI’s GPT-3.5 Turbo and GPT-4 models with fine-tuning and few-shot learning strategies, including a novel few-shot learning strategy for custom-tailored few-shot example selection per request. PheNormGPT was evaluated in the BioCreative VIII Track 3: Genetic Phenotype Extraction from Dysmorphology Physical Examination Entries shared task. PheNormGPT achieved an F1 score of 0.82 for standard matching and 0.72 for exact matching, securing first place for this shared task.

## Introduction

The biomedical Natural Language Processing (NLP) field has witnessed remarkable progress in recent years, driven by the increasing availability of electronic health records (EHRs) and the pressing need for efficient and accurate clinical information extraction. Phenotyping, which is the process of extracting complex trait information from clinical text, is a crucial part of this field that supports a wide range of applications, such as personalized medicine, understanding phenotype–genotype relations, and learning health care systems [1]. Effective and practical data-based phenotyping algorithms can leverage EHRs to improve patient care and health care standards [2]. As such, NLP can support phenotyping by facilitating diagnosis extraction and categorization, phenotype discovery, and drug-related research [3] by processing substantial amounts of unstructured textual data quickly and reliably. Thus, NLP can also play a key role in improving the efficiency and portability of the phenotyping processes [4].

One use of NLP for phenotyping is the task of concept normalization, where phenotypic information found in clinical text is mapped to a standardized vocabulary. The Unified Medical Language System (UMLS) [5] has been a foundational resource for broader biomedical informatics by facilitating the mapping of clinical concepts across different terminologies. However, the Human Phenotype Ontology (HPO) [6] can be utilized as a more specialized framework for normalizing phenotypic information, offering a more focused vocabulary for describing phenotypic abnormalities associated with human diseases. While there is a rich body of literature on the exploration of normalization with UMLS [7–14], the exploration of normalization with HPO is not as vast due to its specialized nature.

A diverse array of NLP methodologies has been applied to phenotypic data normalization. These include rule-based approaches [15–17] and hybrid approaches that combine rule-based approaches with more advanced deep learning and transformers-based approaches [18–20]. The recent emergence of Generative Pre-trained Transformers (GPTs) [21] and other large language models (LLMs) [22, 23] has opened new avenues for NLP research, showing potential in biomedical informatics [24].

This manuscript presents PheNormGPT, a framework for performing normalization of abnormal medical findings in clinical text to HPO. PheNormGPT explores the potential of OpenAI’s GPT-3.5 Turbo and GPT-4 [25] models in the phenotype normalization setting by experimenting with fine-tuning and few-shot learning strategies, supported by prompt engineering and rule-based algorithms. It proposes a novel few-shot example sampling approach that allows for custom-tailored selection of examples for each request. PheNormGPT is developed and evaluated by participating in BioCreative VIII Track 3, a shared task focused on building automated systems that extract and normalize key findings from dysmorphology physical examination entries.

## Methods

### Original dataset

The dataset for developing and evaluating PheNormGPT comes from BioCreative VIII Track 3, a shared task for normalizing key medical findings. The dataset consists of 3136 short, de-identified organ system observations collected from dysmorphology physical examinations of 1652 pediatric patients at the Children’s Hospital of Philadelphia. A medical annotator manually reviewed each observation, mapped the normal and abnormal findings in the text to the best matching HPO concept, and recorded the findings’ boundaries. Examples of how the dysmorphology physical examination observations were annotated can be seen in Table 1.

**Table 1.** Dataset example

Observation ID	Text	HPO ID	Spans	Polarity
D433F04E6AD5E56	EYES: partial synophrys, long lashes, horizontal slant	HP:0000664	14–23	–
D433F04E6AD5E56	EYES: partial synophrys, long lashes, horizontal slant	HP:0000527	25–36	–
8A1EEF66A345576	MOUTH: normal lips, tongue, high palate	HP:0000218	28–39	–
8A1EEF66A345576	MOUTH: normal lips, tongue, high palate	HP:0000159	7–18	X
8A1EEF66A345576	MOUTH: normal lips, tongue, high palate	HP:0000157	7–13, 20–26	X
879246677902DE5	NEUROLOGIC: very active	NA	NA	NA

Collected from *BioCreative—Track 3: Genetic Phenotype Extraction and Normalization from Dysmorphology Physical Examination Entries (genetic conditions in pediatric patients)*. https://biocreative.bioinformatics.udel.edu/tasks/biocreative-viii/track-3/. “NA” indicates data are not available.

The task organizers split this dataset into training, validation, and test sets. The task participants were provided with only the training and the validation sets. During the development of PheNormGPT, the training set was used to fine-tune the GPT models and to sample few-shot examples. Consecutively, the validation set was used to evaluate PheNormGPT, perform error analysis, and make associated improvements to its approaches. Once the development of PheNormGPT was finalized, PheNormGPT was used for inference on the test set using both training and validation sets as training data. The training and validation datasets comprised 2170 observations and 3247 key findings mapped to HPO. A summary of further statistics can be found in Table 2. Since the gold-standard annotations for the test set were not shared with the participants, corresponding annotation-related statistics are not available.

**Table 2.** Dataset statistics

Dataset statistic	Training dataset	Validation dataset	Test dataset
Number of observations	1716	454	966 + 2427 decoys
Number of empty observations	205	49	–
Empty observation rate	12%	11%	–
Number of concepts	2562	685	–
Concepts per observation	1.49	1.51	–
Number of distinct concepts	708	359	–
Observations per distinct concept	2.42	1.26	–
Number of joint spans	2193	589	–
Number of disjoint spans	369	96	–
Disjoint span rate	14%	14%	–
Number of organs/body systems	20	19	20

### Updated dataset

Several updates were applied to the original dataset during the development of PheNormGPT to promote consistency across different observation annotations. There are two primary reasons for making such updates to the original dataset. Firstly, this allowed for increasing consistency for spans annotated for a given concept, which would enhance the performance for predicting spans. For example, there were occasional variances between the inclusion of modifiers such as “mild,” “slightly,” and “prominent.” The first and second rows of Table 3 show one such example, where the annotated entity boundaries are marked with square brackets. These cases were corrected to consistently exclude said modifiers from any entity boundary, as outlined in the task annotation guidelines.

**Table 3.** Dataset inconsistency examples

Inconsistency type	Observation ID	Exact annotated observation	HPO ID
Span	8bb2b415c3263e47b2303bf45efec958	EYES: Prominent [infraorbital creases]	HP:0100876
Span	001d6c29f4e6ab6d37e2a4b0b84db25c	EYES: [Prominent infraorbital creases]	HP:0100876
HPO Concept	5d1c983da5c12cd593edc75ee9548f76	NECK: [Excess nuchal skin]	HP:0005989
HPO Concept	a23e72ed8d70c59e91ad638f4329ff14	NECK: [excess nuchal skin] and	HP:0000474

Secondly, the updates also increase consistency for concepts mapped to a given entity. Such annotation errors may arise from multiple concepts existing in HPO that are close matches for a single entity. These cases were resolved by looking at the entire dataset, picking the most consistent concept assigned to a given entity, and updating all annotations of said entity in the entire dataset. Resolving such inconsistencies also reduces conflicts with PheNormGPT’s dictionary matching approaches, which will be discussed in the following sections. The third and fourth rows of Table 3 show such an example. In this example, HPO ID HP:0005989 corresponds to a concept with the preferred term “redundant neck skin” with a synonym of “excess neck skin,” and HPO ID HP:0000474 corresponds to a concept with the preferred term “thickened nuchal skin fold” with a synonym of “excess nuchal skin.”

Both types of inconsistencies detailed above were detected using automatic methods. Afterward, they were manually reviewed and updated based on similar annotations in the rest of the dataset and annotation guidelines. Such updates allow PheNormGPT to rely better on example-based approaches with LLMs, reducing the possibility of data suggesting inconsistencies across desired annotation behavior.

In the end, 24 spans and 19 HPO concept IDs out of 2770 concept annotations in the training dataset, and 8 spans and 6 HPO concept IDs out of 734 concept annotations in the validation dataset were updated. In addition to this, four concept annotation additions and one concept annotation merge in the training set, and one concept annotation addition and one concept annotation merge in the validation set were performed, where a concept merge entails combining two co-existing concepts HP:0000369 (low-set ears) and HP:0000358 (posteriorly rotated ears) into a single concept HP:0000368 (low-set and posteriorly rotated ears).

Furthermore, 329 concept annotations from the training set and 78 concept annotations from the validation set that recorded normal findings were removed from the dataset to maximize performance for finding abnormal findings. Despite their presence in the dataset, normal findings are not a part of the evaluation process in this shared task, and therefore, they were not utilized to avoid providing undesired information to the models during training.

### Prompt engineering strategy

PheNormGPT relies on LLMs, namely interactions with GPT-3.5 and GPT-4, to extract key findings to normalize from the observation texts. In particular, it uses the snapshot gpt-3.5-turbo-0613 for fine-tuning and the snapshots gpt-3.5-turbo-16k-0613 and gpt-4-0613 for few-shot learning. The prompting strategies used to do so rely on effective and practical expression of information through GPT. PheNormGPT utilizes OpenAI’s ChatCompletion application programming interface (API) with the GPT models to extract named HPO entities from given observations. This setup allows for using three types of messages within a prompt: system message, user message, and assistant message. PheNormGPT utilizes each for a different purpose to best represent the task fitting the standards for LLMs and clearly distinguish each message’s roles. Firstly, it uses the system message to present task definitions and related instructions. Next, it uses the user message to input data for GPT to process, such as an observation text. Finally, it uses the assistant message containing the GPT responses as a list of extracted and normalized entities. Accordingly, PheNormGPT only submits system and user messages in an inference setting and receives outputs from models as an assistant message. A prompt template representation of this strategy is presented in Table 4.

**Table 4.** Prompt template representation

System message	Extract Human Phenotype Ontology (HPO) concepts from the given text of a physical examination note into a table containing standard HPO preferred term and marked original text with square brackets surrounding the words associated with the HPO term.
User message	FACE: elongated face; flattened midface
Assistant message	| HPO Preferred Term | Marked Observation | | long face | FACE: [elongated face]; flattened midface | | midface retrusion | FACE: elongated face; [flattened midface] |

In the prompt template used, the system message is a fixed, short set of instructions. It is the first message in each prompt submitted to GPT. The system message mainly specifies the task’s context and setting rather than providing the complete set of instructions or defining how GPT should behave by itself. Most instructions and specifics about how GPT should behave when extracting entities are conveyed through examples instead. These examples are supplied to GPT using fine-tuning or few-shotting strategies, which will be discussed in the following sections.

Consecutively, the user message consists of a single unannotated observation text from which GPT should extract and normalize key medical findings. In return, the assistant message corresponding to the user message includes relevant information that PheNormGPT would need to facilitate concept extraction and normalization. As a result, GPT has to extract a list of entities, and for each entity, it has to identify a corresponding HPO concept and span of that entity in the observation text. Using this setup, the model would be able to extract a list of entities, along with their corresponding preferred HPO concepts from the observation text.

To satisfy these requirements, PheNormGPT makes GPT return the list of extracted and normalized entities following the format provided in the table within the assistant message. This table includes headers and a row for each entity found in the text given to GPT. It consists of two columns: preferred HPO term and marked observation text. An example of such a table can be seen in Table 4. In the example, the given observation has two extracted entities and two corresponding concepts. For the first concept, the preferred HPO term is “long face,” and the text “elongated face,” the observed term, is marked from the observation via square brackets. For the second concept, the preferred HPO term is “midface retrusion,” and the observed term “flattened midface” is marked in the text accordingly.

The first column, which is the preferred HPO term, is utilized for normalization purposes. This value is a substitute for HPO concept IDs, which can uniquely identify concepts within HPO. However, GPT models are inconsistent when identifying HPO IDs for a given entity. While they correctly identify HPO concept IDs for commonly observed concepts such as hypertension when directly asked, this behavior does not generalize across most other HPO concepts, and they often hallucinate an incorrect HPO concept ID instead. This behavior is shown to be generalized across other types of unique identifiers [26]. Despite this, GPT models demonstrate an understanding of what ontologies are, what HPO is, and approximately what kinds of concepts would be present in HPO. Therefore, instead of requesting a unique identifier code to identify an HPO concept, PheNormGPT requests the preferred HPO term for each entity. This is a roundabout approach for GPT to produce information that can lead to identifying an HPO concept. How PheNormGPT uses the preferred HPO term in normalization will be discussed in the following sections.

The second column, marked text, is utilized primarily for span identification. This column includes the original text from which GPT should extract entities, with one difference: marking of the entity. For each row, this column marks the entity in the original text with square brackets, as demonstrated in Table 4. The spans for each entity can be parsed using this column’s value, where the beginning of the entity span is where the first square bracket starts, and the end of the entity span is where the last square bracket ends. Additionally, this format supports both continuous and discontinuous spans, as disjoint sections can be surrounded with square brackets separately. To further support this format, PheNormGPT pre-processes the input text by replacing all pre-existing square bracket characters with round brackets before sending requests to GPT so that square bracket characters are exclusively used for identifying entity spans.

The marked text approach is an alternative to directly retrieving character offset numbers from the GPT models. However, as a generative model, they are unreliable in returning this information and prone to hallucinating false span offsets. More research in this domain has come to similar conclusions and uses alternative methods to mark text in similar settings, such as marking text with hashtags [27].

### Learning strategies

PheNormGPT heavily relies on examples to guide the GPT model in comprehending the desired output effectively. This is achieved by implementing two different learning strategies for LLMs: fine-tuning and few-shot learning. Fine-tuning involves adjusting the parameters of a pre-trained model specifically for a new task, allowing the model to adapt its knowledge to the nuances of the task at hand. This process requires a dataset related to the target task, through which the model learns the specifics of the task by adjusting its weights accordingly. On the other hand, few-shot learning leverages the model’s existing knowledge and capabilities by providing it with a small number of examples at inference time, demonstrating what is expected of it. This method relies on the model’s ability to generalize from these examples to perform the task without the need for extensive retraining.

### Fine-tuning strategy

**Figure 1.**
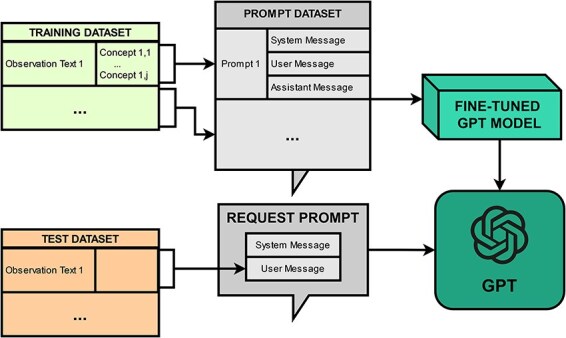
Fine-tuning workflow.

PheNormGPT’s fine-tuning approach was only applied to GPT-3.5-Turbo, as fine-tuning GPT-4 was not available during the development of this framework. Figure 1 presents a visualization of the fine-tuning approach.

The fine-tuning approach consists of creating a prompt dataset and fine-tuning a model via OpenAI API. The prompt dataset consists of one prompt per every observation in the dataset, where each prompt includes three messages, as described in the previous section. Fine-tuning is performed by uploading the prompt dataset and initiating a fine-tuning job through the API. Once a model is fine-tuned, inference is performed on it by submitting prompts consisting of a system and a user message, and receiving an assistant message back that includes a table of entities as previously described, which will be later post-processed for normalization.

### Few-shot learning strategy

**Figure 2.**
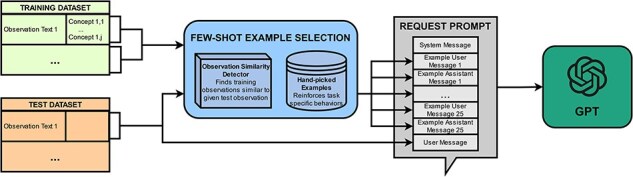
Few-shot learning workflow.

PheNormGPT also experiments with a few-shot learning strategy as an alternative to the fine-tuning strategy. This strategy uses semantically relevant examples to best characterize the behavior desired for each specific example. This is done by utilizing a custom few-shot example selection algorithm, visualized in Fig. 2.

The max token limit for GPT-4 during the development of PheNormGPT allowed for 8k tokens per request. In consideration of this limit, 25 few-shot examples are included per request. These few-shot examples are inserted between the system message and the final user message, where the final user message is the text on which GPT is asked to extract and normalize entities. Each few-shot example consists of a user message, which includes an observation text similar to the final user message, and an assistant message, which includes the table of entities in the corresponding observation text. These 25 few-shot examples are selected in a custom-tailored manner per observation text to perform inference.

PheNormGPT generates a list of few-shot examples using three steps for each request. In the first step, PheNormGPT selects observations from the training set that are most similar to the final observation text of the request and includes at least one concept annotation. spaCy’s document similarity is used as the similarity function, which performs a cosine similarity using an average of word vectors [28]. A total of 15 out of 25 examples are selected using this method.

For the second step, PheNormGPT appends a manually selected list of five examples per body part to include “tricky” examples to the few-shot examples list. These examples mainly aim to guide GPT through BioCreative VIII Track 3’s specific task requirements that are not always followed when described in the system prompt. Such requirements include correctly determining span offsets for examples that require tagging the header body part in each observation or excluding modifiers such as “slightly” or “mild” per annotation guidelines. Table 5 shows examples that would be eligible for this category.

**Table 5.** Examples demonstrating task-specific behavior

Behavior description	Example annotated observation
Disjoint span	EARS: [Low-set] left [ear]
Modifier exclusion	EYES: Mildly [low-set ear]
Organ header annotation	[EAR]: [crumpled]
Organ header annotation	EAR: [crumpled ear]
All of the above	[NOSE]: slightly [bulbous]

In the third step, PheNormGPT adds the five most similar negative examples from the training set to the final observation text of the request to the few-shot examples list. A negative example refers to an observation that does not include any key findings and, therefore, has no entities to extract. This step ensures that GPT understands it has the option to extract no entities if appropriate and shows it the format to use when returning a response with no entities for parsability.

Once PheNormGPT compiles the list of 25 few-shot examples through the three steps, it shuffles the list to randomize the distribution of the examples. Inference is performed by submitting the combined prompt to GPT via OpenAI API and receiving an assistant message back that includes a table of entities, as previously described.

### Concept normalization

**Figure 3.**
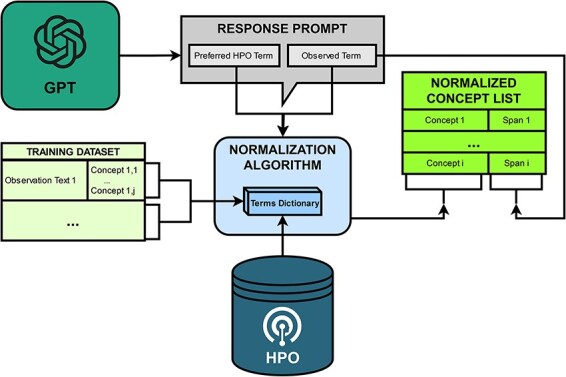
Normalization workflow.

PheNormGPT follows a two-step normalization process for determining HPO concepts matching the entities identified by GPT in the table it returns, visualized in Fig. 3.

PheNormGPT utilizes a dictionary that maps term names to HPO concept IDs for both steps. This dictionary is constructed from the terms and the synonyms from HPO and the annotated terms mapped to HPO concept IDs in the training set. For BioCreative VIII Track 3, 5219 HPO concepts, such as modifier concepts and a list of unobservable concepts shared by the task organizers, are excluded from this dictionary. Additionally, stemming and case normalization are applied when attempting a match with this dictionary to increase dictionary matching performance.

**Figure 4.**
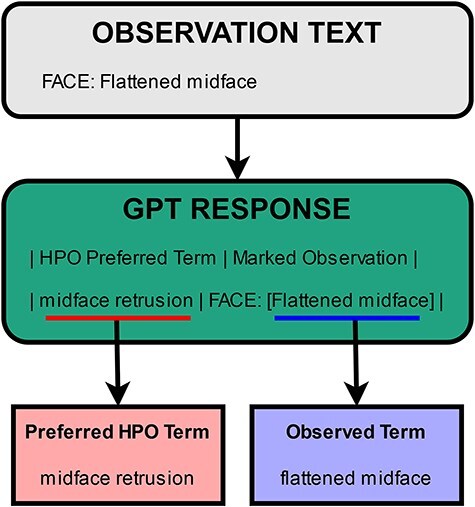
Illustration of terms extracted from the GPT response.

As mentioned in the previous sections, the table returned by GPT includes two terms to potentially normalize per entity. The first one is the returned preferred HPO term. The second one is the observed term, which is the form in which the entity is present in within the given text. In other words, it is the mention of that entity in text, or the term marked using the square brackets. An example illustrating the two terms is presented in Fig. 4. In the first step of the normalization process, PheNormGPT tries to normalize the observed term as it appears in the text via dictionary matching. If there is a match, the entity is assigned the matching HPO concept, and the process is complete. Otherwise, PheNormGPT moves to the second step of the normalization process. In the second step, it tries to normalize the preferred HPO term returned by GPT, using dictionary matching again. If there is a match, the entity is assigned the matching HPO concept, and the process is complete. Otherwise, PheNormGPT fails to normalize that entity and does not record any HPO concepts for it.

In summary, the first step involves simple dictionary matching using observed terms through the dataset and known terms through HPO, while the second step employs dictionary matching using preferred terms recommended by GPT.

## Results

In the BioCreative VIII Track 3 evaluation step, PheNormGPT was evaluated on the test corpus of the shared task. For this step, task organizers only shared the observation texts with the participants and did not include the gold-standard annotations. The evaluation was conducted across three settings. The normalization only setting checked the performance of HPO identifier prediction. The strict extraction and overlapping extraction settings checked both the performance of HPO identifier prediction and entity boundary overlaps via strict and overlapping matches, respectively. Across multiple submissions, PheNormGPT received a maximum F1 score of 0.82 in the normalization only setting, and a maximum F1 score of 0.72 in the strict extraction and normalization setting.

Submissions to the test set evaluation and their scores are listed in Table 6. These submissions combine various techniques described in the “Methods” section and experiment with different source models, including GPT-3.5 Turbo and GPT-4, as well as learning techniques such as fine-tuning and few-shot learning. A combination of GPT-4 and fine-tuning is not present due to the lack of GPT-4 fine-tuning availability via OpenAI API during the development. The role of the normalization targets is also explored, where each option is attempted with normalization of both the observed term and the predicted preferred HPO term, and with normalization of only the observed term.

**Table 6.** Performance on test set

			Normalization only			Overlapping extraction and normalization			Strict extraction and normalization		
Model	Learning technique	Normalization target	P	R	F1	P	R	F1	P	R	F1
GPT-3.5 Turbo	Fine-tuning	Observed term and predicted preferred term	0.8142	0.7488	0.7801	0.8134	0.7448	0.7776	0.7909	0.6463	0.7113
GPT-3.5 Turbo	Fine-tuning	Only observed term	0.9129	0.5747	0.7054	0.9127	0.5731	0.7041	0.9078	0.5397	0.6770
GPT-3.5 Turbo	Few-shot Learning	Observed term and predicted preferred term	0.8432	0.6884	0.7580	0.8426	0.6852	0.7558	0.8041	0.5254	0.6356
GPT-3.5 Turbo	Few-shot Learning	Only observed term	0.9103	0.4921	0.6388	0.9100	0.4905	0.6374	0.9016	0.4444	0.5953
GPT-4	Few-shot Learning	Observed term and predicted preferred term	0.8417	0.7989	0.8197	0.8409	0.7941	0.8168	0.8104	0.6423	0.7166
GPT-4	Few-shot Learning	Only observed term	0.9363	0.5604	0.7011	0.9361	0.5588	0.6999	0.9313	0.5175	0.6653

Overall, few-shot learning with GPT-4 has achieved the highest F1 score, relying on both the normalization of the observed term and the normalization of the preferred term returned by GPT. Additionally, using the observed term alone results in higher precision at the cost of lower recall, which is expected as such strategies do not utilize GPT models’ preferred term prediction capabilities, which can introduce false predictions.

## Discussion

As observed in the dataset updates section and the few-shot learning approach, normalization with GPT models can lead to various extraction errors and inconsistencies for multiple types of behaviors. These errors and inconsistencies were observed while evaluating the model using the validation set during the preliminary stages of PheNormGPT’s development. They encompass inaccurate span boundaries and imprecise normalization, appearing in various forms across multiple examples within all subsets of the original dataset.

First, boundary and normalization errors were observed for entities present in the training set. Such errors occasionally originate from the dataset itself, as similar behavior is demonstrated in the original dataset with variations. To remedy this behavior and ensure the model primarily follows the annotation guidelines specified in the shared task, previously described updates were applied to the provided dataset.

Another source of boundary errors corresponded to GPT models failing to follow task-specific behavior, particularly concerning disjoint spans and boundaries, including organ headers. Corrections to such behaviors are enforced via fine-tuning or few-shot learning. To ensure these behaviors are well-presented in the few-shot learning setting, a manually selected list of examples that demonstrate such behaviors are consistently included in the few-shot examples generated for inference, as discussed in the earlier sections.

One other source of errors in PheNormGPT concerns the marked text approach. Occasionally, GPT models return a slightly different text in the marked-text field than the one provided in the request. These differences are primarily the result of GPT fixing typos in the original text, such as misspelled words or missing spaces. Additionally, the presence of special characters such as “+” can cause such errors. For example, GPT models were observed to modify the input “HEAD: +cephalohematoma” to “HEAD: +cephalohematoma+” during earlier development iterations. This behavior causes complexities when parsing the character offsets and reduces the accuracy of this method for such rare cases. On occasions where an extracted concept has span offsets that would be out of bounds for the original observation text, PheNormGPT discards the extracted concept to avoid boundary errors. Future approaches can overcome this issue by expanding post-processing strategies or experimenting with prompt engineering to prevent it.

A potential area of improvement when using LLMs in this context is to ask LLMs to double-check their responses and self-correct. This was shown to increase performance in other biomedical informatics settings [29]. However, this approach doubles the time and the cost required for inference due to each request being made at least twice. Therefore, it was not a part of the experimentations for PheNormGPT.

Finally, PheNormGPT is built around OpenAI’s GPT models, which pose their own considerations. OpenAI’s API-based consumption model means that the models are deployed on OpenAI servers, and input and output data must also go through their servers. This can create concerns about data privacy and means that the approach presented in this paper is not directly applicable to non-de-identified data. This creates future research opportunities for extending PheNormGPT’s approaches to other open-source LLMs, such as LLaMA [22] and Falcon [23].

## Conclusion

This manuscript introduces PheNormGPT, a framework for extracting and normalizing medical key findings to HPO, utilizing OpenAI’s GPT models in two separate approaches, combining rule-based methods, LLMs, and prompt engineering strategies. PheNormGPT is developed and evaluated on the dataset provided by BioCreative VIII Track 3 and has achieved the highest performance among the shared-task participants, highlighting the potential of LLMs in phenotypic data normalization. PheNormGPT’s approaches include a normalization prompting strategy harnessing the strengths of LLMs and requesting relevant information in a roundabout way to reduce hallucinations, and a few-shot learning algorithm that generates a specialized prompt with examples that demonstrate desired behavior in a custom and focused manner for each input. While the full potential of LLMs is not yet explored, and there may be higher performing prompting strategies in this area, PheNormGPT shows that LLMs can play a significant role in future research in this domain.

## Data Availability

The training and validation data underlying this article are available on GitHub at https://github.com/Ian-Campbell-Lab/Clinical-Genetics-Training-Data/.
